# Vonoprazan-Based Third-Line Therapy Has a Higher Eradication Rate against Sitafloxacin-Resistant *Helicobacter pylori*

**DOI:** 10.3390/cancers11010116

**Published:** 2019-01-19

**Authors:** Yoshimasa Saito, Kaho Konno, Moeka Sato, Masaru Nakano, Yukako Kato, Hidetsugu Saito, Hiroshi Serizawa

**Affiliations:** 1 Department of Gastroenterology and Hepatology, Kitasato University Kitasato Institute Hospital, 5-9-1 Shirokane, Minato-ku, Tokyo 108-8642, Japan; msnakano@insti.kitasato-u.ac.jp (M.N.); yukako5380@gmail.com (Y.K.); 2 Division of Pharmacotherapeutics, Keio University Faculty of Pharmacy, 1-5-30 Shibakoen, Minato-ku, Tokyo 105-8512, Japan; konno-kaho.1013@a2.keio.jp (K.K.); moeka9494@gmail.com (M.S.); saito-hd@pha.keio.ac.jp (H.S.); 3 Healthcare Department, Hitachi Systems, Ltd. 1-2-1 Osaki, Shinagawa-ku, Tokyo 141-8672, Japan; serizawa-h@jcom.zaq.ne.jp

**Keywords:** *Helicobacter pylori*, vonoprazan, esomeprazole, proton pump inhibitor, clarithromycin, sitafloxacin

## Abstract

Eradication of *Helicobacter pylori* (*H. pylori*) is an effective strategy for preventing various gastrointestinal diseases such as gastric cancer and mucosa-associated lymphoid tissue (MALT) lymphoma. However, the eradication success rate is decreasing because of a recent increase in drug-resistant strains of *H. pylori*. Here, we evaluated the success rate of eradication therapy with vonoprazan (VPZ), a new potassium-competitive acid blocker, against drug-resistant *H. pylori*. In total, 793 patients who received *H. pylori* eradication therapy were investigated retrospectively. All underwent esomeprazole (EPZ)-based triple therapy (*n* = 386) or VPZ-based triple therapy (*n* = 407) for first-, second- and third-line *H. pylori* eradication for 7 days. The overall success rates of first- and third-line *H. pylori* eradication were significantly higher for VPZ-based triple therapy (88.4% and 93.0%, respectively, per protocol (PP)) than for EPZ-based triple therapy (69.5% and 56.5%, respectively, PP). Moreover, the success rates of first- and third-line eradication of clarithromycin (CLR)- and sitafloxacin (STFX)-resistant *H. pylori* were significantly higher for VPZ-based triple therapy (72.0% and 91.7%, PP) than for EPZ-based triple therapy (38.5% and 20.0%, PP). In addition, patient age did not affect the eradication rate of VPZ-based first-line therapy, whereas the success rate of EPZ-based therapy was lower in patients under 65 years of age. Our results clearly demonstrated that VPZ-based therapy achieved a higher eradication rate even against CLR- and STFX-resistant *H. pylori*, and that patient age did not affect the eradication rate of VPZ-based therapy. These findings suggest that dual therapy using VPZ and amoxicillin may be sufficient for standard *H. pylori* eradication, and may thus also be beneficial for avoiding antibiotic misuse.

## 1. Introduction

*Helicobacter pylori* (*H. pylori*) is a common bacterium that causes upper gastrointestinal disorders such as atrophic gastritis, peptic ulcers, functional dyspepsia (FD), gastric mucosa-associated lymphoid tissue (MALT) lymphoma, and gastric cancer [1,2,3,4,5]. Eradication of *H. pylori* is an effective strategy for preventing such diseases [6,7,8]. In particular, Fukase et al. [5] performed a multi-centre, open-label, randomised controlled trial to investigate the prophylactic effect of *H. pylori* eradication on the development of metachronous gastric carcinoma after endoscopic resection for early gastric cancer. They clearly demonstrated that prophylactic eradication of *H. pylori* is beneficial for the prevention of gastric cancer [5]. Establishment of the optimal regimen for *H. pylori* eradication therapy is very important for the prevention of gastric cancer. Recently, however, the success rate of the first-line eradication regimen comprising clarithromycin (CLR) combined with amoxicillin (AMX) and traditional proton pump inhibitors (PPIs) has dropped due to an increase in *H. pylori* strains that are resistant to CLR.

As antibiotics are more stable in higher-pH gastric environments, strong gastric acid inhibition increases the success rate of *H. pylori* eradication. Recently, vonoprazan (VPZ), a new potassium-competitive acid blocker (P-CAB), was approved for use in *H. pylori* eradication therapy in Japan, the Philippines, and Singapore. VPZ shows a more potent and sustained acid-inhibitory effect than other conventional PPIs. Recent studies have reported the superiority of VPZ-based triple therapy over conventional PPI-based triple therapy for first-line *H. pylori* eradication [9,10,11]. However, it remains unclear whether VPZ-based therapy is effective against drug-resistant *H. pylori* in the context of third-line eradication. In the present study, we compared the success rate of VPZ-based triple therapy with esomeprazole (EPZ)-based triple therapy for first-, second-, and third-line *H. pylori* eradication. We also investigated the effect of VPZ-based triple eradication therapy against CLR- and sitafloxacin (STFX)-resistant *H. pylori*.

## 2. Methods

### 2.1. Patients and Study Design

In total, 793 patients who were diagnosed as positive for *H. pylori* infection and received *H. pylori* eradication therapy between January 2013 and February 2018 at Kitasato Institute Hospital (Tokyo, Japan) were investigated retrospectively (Table 1). Diagnosis of *H. pylori* infection was performed by the ^13^C-urea breath test (UBT) or endoscopic biopsy-based test (i.e., histological examination and *H. pylori* culture). These patients received EPZ-based triple therapy (*n* = 386) or VPZ-based triple therapy (*n* = 407) for first-, second-, or third-line *H. pylori* eradication (Table 1). The regimens of these therapies were as follows.

Conditions for these therapies: First-line triple therapy: EPZ 40 mg/day or VPZ 40 mg/day, AMX 1500 mg/day, and CLR 400 mg/day for 7 days; Second-line triple therapy: EPZ 40 mg/day or VPZ 40 mg/day, AMX 1500 mg/day, and metronidazole (MTZ) 500 mg/day for 7 days; Third-line triple therapy: EPZ 40 mg/day or VPZ 40 mg/day, AMX 1500 mg/day, and STFX 200 mg/day for 7 days.

All drugs were given twice per day. Three months after eradication, the presence of *H. pylori* infection was investigated by UBT. To evaluate the susceptibility of *H. pylori* to the antimicrobials used, the minimum inhibitory concentrations (MIC) of CLR, MTZ, and STFX were examined. The MIC values of CLR and MTZ for *H. pylori* resistance were determined as ≥1 µg/mL and ≥15 µg/mL based on the European Committee on Antimicrobial Susceptibility Testing (EUCAST) Clinical Breakpoint [12]. The MIC value of STFX for *H. pylori* resistance was determined as ≥ 0.12 µg/mL according to recent studies [13,14]. In the present study, we did not find AMX-resistant *H. pylori* in patients who received eradication therapy.

The study was reviewed and approved by the institutional review board of the Kitasato Institute Hospital. The ethical codes related to this research are 16032, 16033, and 16034, approved on 13 February 2018.

### 2.2. Statistics

The *H. pylori* eradication rate was evaluated in terms of intention-to-treat (ITT) and per protocol (PP). Patients who did not return to the hospital for UBT three months after eradication therapy were excluded from PP analysis. Data were analysed by chi-squared test, and differences at *p* < 0.05 were considered significant.

## 3. Results

### 3.1. Overall Success Rates of First- and Third-Line H. Pylori Eradication Are Significantly Higher for VPZ-Based Therapy Than for EPZ-Based Triple Therapy

**Figure 1:**
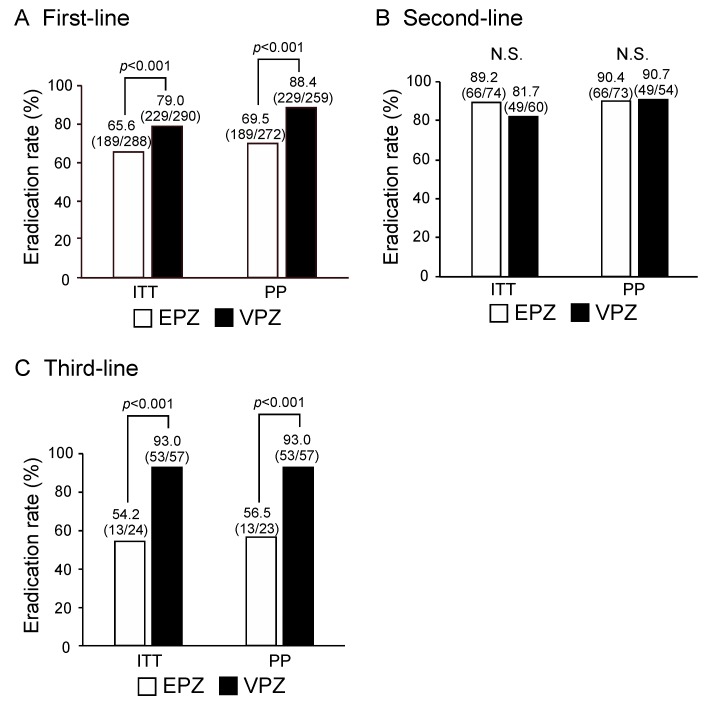
The overall success rates for first-, second-, and third-line *H. pylori* eradication between VPZ-based triple therapy and EPZ-based triple therapy. (**A**) The first-line eradication rates for VPZ-based triple therapy evaluated by intention-to-treat (ITT) and per protocol (PP) (79.0% and 88.4%, respectively) were significantly higher than those for EPZ-based triple therapy (65.6%, *p* < 0.001 and 69.5%, *p* < 0.001, respectively). (**B**) There was no significant difference between the second-line eradication rates for vonoprazan (VPZ)-based triple therapy (81.7% and 90.7%, respectively) and esomeprazole (EPZ)-based triple therapy (89.2% and 90.4%, respectively). N.S.: not significant. (**C**) The third-line eradication rates for VPZ-based triple therapy including STFX (93.0% and 93.0%, respectively) were significantly higher than those for EPZ-based triple therapy including STFX (54.2%, *p* < 0.001 and 56.5%, *p* < 0.001, respectively).

First, we compared the overall success rates for first-, second-, and third-line *H. pylori* eradication between VPZ-based triple therapy and EPZ-based triple therapy. As shown in Figure 1A, the first-line eradication rates for VPZ-based triple therapy evaluated by ITT and PP (79.0% and 88.4%, respectively) were significantly higher than those for EPZ-based triple therapy (65.6%, *p* < 0.001 and 69.5%, *p* < 0.001, respectively). On the other hand, there was no significant difference between the second-line eradication rates for VPZ-based triple therapy (81.7% and 90.7%, respectively) and EPZ-based triple therapy (89.2% and 90.4%, respectively, Figure 1B). The third-line eradication rates for VPZ-based triple therapy including STFX (93.0% and 93.0%, respectively) were significantly higher than those for EPZ-based triple therapy including STFX (54.2%, *p* < 0.001 and 56.5%, *p* < 0.001, respectively, Figure 1C). Thus, 7-day VPZ-based triple therapy was more effective than 7-day EPZ-based triple therapy for first- and third-line *H. pylori* eradication.

### 3.2. Success Rates for First- and Third-Line Eradication of CLR-Resistant H. Pylori Are Significantly Higher for VPZ-Based Therapy Than for EPZ-Based Triple Therapy

Given that the success of *H. pylori* eradication therapy is affected by the susceptibility of *H. pylori* to the antibiotics used, we compared the success rates (PP) of first- and third-line eradication for drug-susceptible or resistant *H. pylori* between VPZ-based triple therapy and EPZ-based triple therapy.

**Figure 2:**
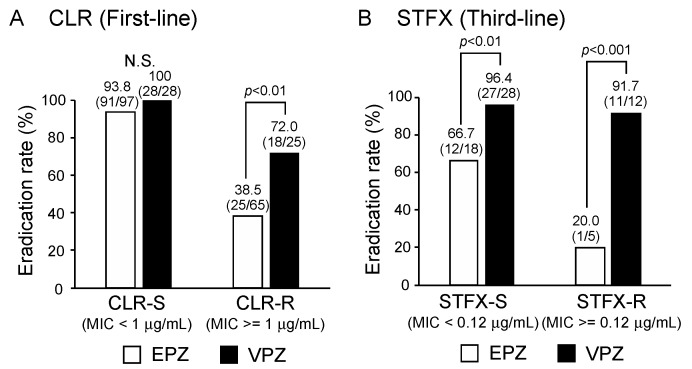
The success rates (PP) of first- and third-line eradication for drug-susceptible or resistant *H. pylori* with VPZ-based triple therapy and EPZ-based triple therapy. (**A**) The first-line eradication rate for CLR-resistant *H. pylori* (minimum inhibitory concentration, minimum inhibitory concentrations (MIC) ≥ 1 µg/mL) was significantly higher for VPZ-based triple therapy (72.0%) than for EPZ-based triple therapy (38.5%, *p* < 0.01), whereas there was no significant difference in the first-line eradication rate for CLR-susceptible *H. pylori* (MIC < 1 µg/mL). (**B**) The third-line eradication rate for STFX-resistant *H. pylori* (MIC ≥ 0.12 µg/mL) was significantly higher for VPZ-based triple therapy (91.7%) than for EPZ-based triple therapy (20.0%, *p* < 0.001). The third-line eradication rate for STFX-susceptible *H. pylori* (MIC < 0.12 µg/mL) was also significantly higher for VPZ-based triple therapy (96.4%) than for EPZ-based triple therapy (66.7%, *p* < 0.01).

As shown in Figure 2A, the first-line eradication rate for CLR-resistant *H. pylori* (MIC ≥ 1 µg/mL) was significantly higher for VPZ-based triple therapy (72.0%) than for EPZ-based triple therapy (38.5%, *p* < 0.01), whereas there was no significant difference in the first-line eradication rate for CLR-susceptible *H. pylori* (MIC < 1 µg/mL).

The third-line eradication rate for STFX-resistant *H. pylori* (MIC ≥ 0.12 µg/mL) was significantly higher for VPZ-based triple therapy (91.7%) than for EPZ-based triple therapy (20.0%, *p* < 0.001) as shown in Figure 2B. Interestingly, the third-line eradication rate for STFX-susceptible *H. pylori* (MIC < 0.12 µg/mL) was also significantly higher for VPZ-based triple therapy (96.4%) than for EPZ-based triple therapy (66.7%, *p* < 0.01). These findings demonstrated that VPZ-based eradication therapy was more effective than EPZ-based eradication therapy, even against CLR- and STFX-resistant *H. pylori.*

### 3.3. Patient Age Does Not Affect the Eradication Rate Achieved by VPZ-Based First-Line Therapy, Whereas That for EPZ-Based Therapy Is Lower in Patients under 65 Years of Age

**Figure 3:**
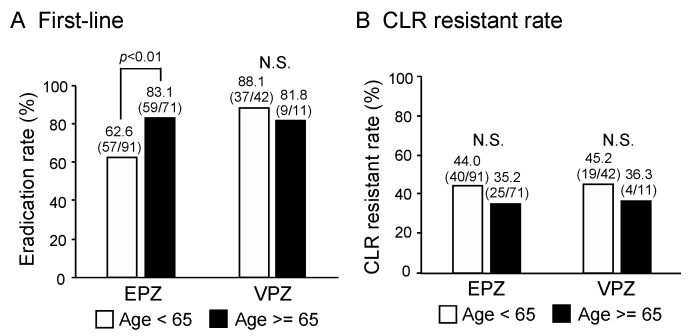
The effect of patient age on the success rate (PP) of first-line *H. pylori* eradication. (**A**) For EPZ-based triple therapy, the success rate of first-line *H. pylori* eradication was significantly higher in patients aged over 65 years than in those under 65 years of age. For VPZ-based triple therapy, there was no significant difference between patients under and over 65 years of age in the success rate of first-line *H. pylori* eradication therapy. (**B**) There was no significant difference in the rate of CLR resistance between patients under and over 65 years of age who received EPZ-based and VPZ-based first-line triple therapy.

The incidence of CLR resistance is increasing, probably due to misuse of CLR in the young and middle-aged populations. Therefore, we examined the effect of patient age on the success rate (PP) of first-line *H. pylori* eradication. As shown in Figure 3A, for EPZ-based triple therapy, the success rate of first-line *H. pylori* eradication was significantly higher in patients aged over 65 years (83.1%) than in those under 65 years of age (62.6%, *p* < 0.01). On the other hand, for VPZ-based triple therapy, there was no significant difference between patients under and over 65 years of age in the success rate of first-line *H. pylori* eradication therapy.

We also compared the rate of CLR resistance between patients below and above 65 years of age who received EPZ-based or VPZ-based first-line triple therapy (Figure 3B). As shown in Figure 3B, there was no significant difference in the rate of CLR resistance between patients under and over 65 years of age who received EPZ-based and VPZ-based first-line triple therapy. Thus, patient age did not affect the eradication rate achieved by VPZ-based first-line therapy, whereas that of EPZ-based therapy was decreased in patients under 65 years of age.

## 4. Discussion

Establishment of the optimal regimen for *H. pylori* eradication therapy is very important for the prevention of gastric cancer. P-CABs are a new class of drug that inhibits gastric hydrogen/potassium-ATPase in a potassium-competitive and reversible manner. VPZ is a novel, orally active P-CAB with a potent and long-lasting anti-secretory effect due to its high accumulation and slow clearance from gastric tissue [15]. The acid-inhibitory effects of VPZ are more potent than those of conventional PPIs including EPZ. In fact, recent studies have shown that VPZ-based triple therapy is more effective than conventional PPI-based triple therapy for first-line *H. pylori* eradication [9,10,11].

Our results have also demonstrated the superiority of VPZ-based triple therapy over EPZ-based triple therapy for first-line eradication of *H. pylori*. The eradication rate achieved with VPZ-based triple therapy for first-line eradication of CLR-resistant *H. pylori* was significantly higher than that for EPZ-based triple therapy. For second-line *H. pylori* eradication, there was no significant difference between VPZ-based and EPZ-based triple therapy, both showing a high eradication rate as described previously [16,17]. It has been reported that the resistance rate of MTZ to *H. pylori* is relatively low (0–4%) in Japan [18]. In the present study, we did not find MTZ-resistant *H. pylori* in patients who received VPZ-based second-line therapy. Thus, we speculate that acid suppression by either VPZ or EPZ did not affect *H. pylori* eradication rates.

Here, we have demonstrated that VPZ-based triple therapy was also more effective than EPZ-based triple therapy for third-line *H. pylori* eradication using STFX. A recent study has shown that the eradication rate of VPZ-based third-line therapy using STFX was significantly higher than that of PPI (rabeprazole or lansoprazole)-based therapy (83.3% vs. 57.1%, PP), which is similar to our results in the present study [19]. Surprisingly, in this context, VPZ-based triple therapy achieved a high eradication rate (91.7%) even for STFX-resistant *H. pylori*, whereas the eradication rate of EPZ-based third-line therapy was low. Marcus et al. have demonstrated that AMX exhibits bactericidal effects on *H. pylori* growth at pH 4.5 and 7.4, but not at pH 3.0 [20]. *H. pylori* may become more susceptible to AMX in higher-pH gastric environments achieved by the potent acid-inhibitor VPZ, which may result in a higher eradication rate of VPZ-based triple therapy even for STFX-resistant *H. pylori*.

The eradication success rate for conventional PPI-based eradication therapy has been decreasing because of the recent increase in drug-resistant *H. pylori* strains. Our results also showed that the rate of STFX-resistant *H. pylori* was high (17/63: 27%). This may be caused by the increasing use of quinolone antibacterial agents including STFX for treatment of various infectious diseases such as respiratory infection and urologic infection. These findings suggest that dual therapy using VPZ and AMX may be sufficient for standard *H. pylori* eradication, and would also be beneficial for avoiding antibiotic misuse. Further studies including comparisons between VPZ-based triple therapy and dual therapy will be necessary for better optimization of VPZ-based *H. pylori* eradication therapy.

Recent studies have reported that first-line *H. pylori* eradication using PPIs fails more frequently in young and middle-aged patients as compared to older patients [21,22]. Our present results showed that the success rate of EPZ-based first-line eradication was significantly higher in patients over 65 years old than in those under 65 years of age. There was no significant difference in the CLR resistance rate between patients under and over 65 years of age who received EPZ-based and VPZ-based first-line triple therapy. The gastric mucosa becomes more atrophic as patients age. This means that older patients usually have gastric acid hyposecretion, which may result in a higher success rate of PPI-based first-line eradication in patients older than 65 years. On the other hand, VPZ-based first-line therapy was independent of patient age and achieved a higher eradication rate even in younger patients, probably because of the potent gastric acid-inhibitory effect of VPZ.

## 5. Conclusions

In conclusion, our present results have clearly demonstrated that VPZ-based therapy achieves a higher eradication rate even against CLR- and STFX-resistant *H. pylori* and that patient age does not affect the eradication rate of VPZ-based therapy. These findings suggest that dual therapy using VPZ and AMX may be sufficient for standard *H. pylori* eradication, and would also be beneficial for avoiding antibiotic misuse. Further studies including comparisons between VPZ-based triple therapy and dual therapy will be necessary for better optimization of VPZ-based *H. pylori* eradication therapy.

## Figures and Tables

**Table 1:** Information of patients who received *Helicobacter pylori* eradication therapy. EPZ: esomeprazole; AMX: amoxicillin; CLR: clarithromycin; VPZ: vonoprazan; MTZ: metronidazole; STFX: sitafloxacin.

Subject	First-Line Triple Therapy		Second-Line Triple Therapy		Third-Line Triple Therapy	
Regimen	EPZ/AMX/CLR (*n* = 288)	VPZ/AMX/CLR (*n* = 290)	EPZ/AMX/MTZ (*n* = 74)	VPZ/AMX/MTZ (*n* = 60)	EPZ/AMX/STFX (*n* = 24)	VPZ/AMX/STFX (*n* = 57)
Age (mean ± SD)	57.9 ± 12.2	60.2 ± 12.6	56.1 ± 13.0	58.3 ± 11.9	48.3 ± 9.88	50.7 ± 12.1
Gender (male/female)	160/128	175/115	36/38	30/30	16/8	25/32
